# Factors associated with self-rated health in Black Canadians: A cross-sectional study

**DOI:** 10.17269/s41997-024-00973-8

**Published:** 2024-11-26

**Authors:** Sheila A. Boamah, Roger Antabe, Shamara Baidoobonso, Josephine Etowa, Pascal Djiadeu, Clemence Ongolo-Zogo, Winston Husbands, Lawrence Mbuagbaw

**Affiliations:** 1 School of Nursing, Faculty of Health Sciences, McMaster University, Hamilton, ON Canada; 2 Department of Health and Society, University of Toronto Scarborough, Toronto, ON Canada; 3 Department of Community Health and Epidemiology, Dalhousie University, Halifax, NS Canada; 4 School of Nursing, Faculty of Health Sciences, University of Ottawa, Ottawa, ON Canada; 5 Department of Health Research Methods, Evidence and Impact, McMaster University, Hamilton, ON Canada; 6 Dalla Lana School of Public Health, University of Toronto, Toronto, ON Canada; 7 Biostatistics Unit, Father Sean O’Sullivan Research Centre, St. Joseph’s Healthcare, Hamilton, ON Canada

**Keywords:** Black Canadians, Self-rated health, Quality of life, Health equity, Canadiens Noirs, Santé auto-évaluée, Qualité de vie, Équité en santé

## Abstract

**Objectives:**

Self-rated health (SRH) has shown to be a strong predictor of morbidity, functional decline, and mortality outcomes. This paper investigates the association between sociodemographic variables (e.g., employment, education, sex) and SRH among Black Canadians.

**Methods:**

We used cross-sectional survey data (*n* = 1380) from the A/C (African Caribbean) Study of first- and second-generation Black Canadians in Toronto and Ottawa. Participants were invited to complete an electronic survey questionnaire in English or French in 2018–2019. Generalized linear model analyses were used to evaluate the associations among sociodemographic factors and self-rated quality of health.

**Results:**

A total of 1380 self-identified Black individuals completed the survey and were included in the analysis. The majority of participants were under the age of 60 (89.7%), female (63.4%), born outside of Canada (75.1%), and residing in Toronto, Ontario (61.9%). The strongest association with poor SRH was found for difficulties accessing health care, sexual orientation, and substance misuse/disorder, while accessing/meeting basic needs was associated with better SRH, following adjustment for other socioeconomic conditions and lifestyle factors.

**Conclusion:**

Our findings underscore the importance of improving the social determinants of health as a conduit to improving the general health status and the quality of life of Black Canadians. Results revealed that Black Canadians may be demonstrating high levels of resilience in circumventing their current social circumstances and structural disadvantages to live the best quality of life. Understanding sociodemographic and socio-structural barriers that Black people face is essential to reducing vulnerabilities to poor outcomes and improving their health and well-being.

## Résumé

**Objectifs:**

L’autoévaluation de la santé s’est révélée être un puissant prédicteur de la morbidité, du déclin fonctionnel et de la mortalité. Cet article étudie l’association entre les variables sociodémographiques (par exemple, l’emploi, l’éducation, le sexe) et l’autoévaluation de la santé chez les Canadiens Noirs.

**Méthodes:**

Nous avons utilisé les données d’une enquête transversale (*n* = 1380) de l’étude A/C (Africain Caribéen) sur les Canadiens Noirs de première et de deuxième génération à Toronto et à Ottawa. Les participants ont été invités à remplir un questionnaire d’enquête électronique en anglais ou en français en 2018‒2019. Des analyses par modèle linéaire généralisé ont été utilisées pour évaluer les associations entre les facteurs sociodémographiques et la qualité de la santé auto-évaluée.

**Résultats:**

Au total, 1380 personnes s’identifiant comme Noirs ont répondu au sondage et ont été incluses dans l’analyse. La majorité des participants avaient moins de 60 ans (89,7%), étaient des femmes (63,4%), étaient nés à l’étranger (75,1%), et résidaient à Toronto, en Ontario (61,9%). Après ajustement pour d’autres conditions socio-économiques et facteurs liés au mode de vie, l’association la plus forte avec un mauvais état de santé auto-évalué a été observée pour les difficultés d’accès aux soins de santé, l’orientation sexuelle et les troubles ou abus de substances, tandis que l’accès aux besoins fondamentaux a été associé à un meilleur état de santé auto-évalué.

**Conclusion:**

Nos résultats soulignent l’importance d’améliorer les déterminants sociaux de la santé comme moyen d’améliorer l’état de santé général et la qualité de vie des Canadiens Noirs. L’accès aux soins de santé et aux besoins fondamentaux sont des prédicteurs importants de la qualité de la santé auto-évaluée. Nos résultats révèlent que les Canadiens Noirs peuvent faire preuve d’une grande résilience en contournant leur situation sociale actuelle et leurs désavantages structurels pour jouir d’une qualité de vie optimale. Il est essentiel de comprendre les obstacles sociodémographiques et socio-structurels auxquels les Noirs sont confrontés, pour réduire leur vulnérabilité aux mauvais résultats et améliorer leur santé et leur bien-être.

## Introduction

In Canada, studies have alluded to the impact of structural racism and interpersonal experiences of racism faced by Black people on their high exposure/susceptibility to poor health outcomes relative to other Canadians (Fante-Coleman et al., 2022; Husbands et al., 2022). These experiences may not only influence how Black people view their health status but also serve to undermine their quality of life—defined as the extent to which an individual’s satisfaction with their overall life circumstances is achieved (Du Mont & Forte, 2016; Husbands et al., 2022; Stevenson, 2020). Anti-Black racism works through social, demographic, economic, and health-related factors to reduce the quality of life of Black Canadians by denying them access to health-enabling resources (DasGupta et al., 2020). Their experiences with everyday racism contribute to an increased susceptibility to chronic health conditions, such as diabetes and hypertension (Haile, 2014; Veenstra & Patterson, 2016). Furthermore, the lack of cultural and structural competence in Canada’s health care system implies that the health needs of Black people are peripheral to the design and delivery of health services. At health services outlets, the specific health care needs of Black Canadians are either neglected or treated as an appendage to the existing services (Dryden & Nnorom, 2021; Fante-Coleman et al., 2022). Consequently, Black Canadians may not only feel alienated and unwelcome in health care spaces but may also be more likely to report unmet health care needs (Etowa et al., 2021; Public Health Agency of Canada, 2020).

As scholarly discourses gradually unveil the structural disadvantages confronting Black people in Canada, rooted in their social, demographic, and economic conditions, limited studies have examined how Black people perceive these attributes to be impacting their health. Specifically, we have limited knowledge of whether or how social, demographic, and economic factors, alongside other health-related factors, are associated with how Black Canadians self-assess their general health status. Only a few prior studies have focused on understanding the differences in self-assessed general health status between Black Canadians and other racial groups. For instance, while the Pan-Canadian Health Inequalities Reporting Initiative suggests that 13% of Black Canadians self-assessed their health to be fair or poor in 2020, the report did not state whether or how this was impacted by demographic, socioeconomic, and health-related factors (Pan-Canadian Health Inequalities Data Tool, 2020).

The aim of this study was to empirically test the influence of sociodemographic variables (e.g., employment, sex) on the overall general state of health among Black Canadians. Using the context of Toronto and Ottawa, two of Canada’s largest cities with a large concentration of Black population, we examined self-assessed general health status among Black Canadians living in the two cities and whether this is shaped by demographic, socioeconomic, and health-related factors.

### Self-rated general health status

Self-rated health (or self-assessed general health) status is a subjective measure of an individual’s own overall health, integrating biological, mental, social, and functional aspects. A series of national and international analyses have consistently demonstrated that self-rated health (SRH) serves as a reliable predictor of mortality (Benjamins et al., 2004; Idler & Benyamini, 1997; Jylha, 2009). Research has identified demographic, socioeconomic, and geographical factors that tend to influence people’s self-assessment of their general health. Notably, a persistent gender gap has been observed where women are more inclined than men to report poorer health status. In Canada, for instance, while 10% of Black men perceived their health as fair or poor, this increased to almost 16% for Black women (Pan-Canadian Health Inequalities Data Tool, 2020). Some authors suggest the gender gap may be due to a combination of biological factors and gender inequalities that disproportionately affect women’s health (Boerma et al., 2016). For socioeconomic factors, higher educational attainment is linked to improved self-assessed health. This is because education equips individuals with the requisite knowledge for a healthy lifestyle and fosters interactive and critical health literacy, enabling them to process health information and navigate complex health systems (Schellekens & Ziv, 2020). Similarly, those from high-income households have improved access to health-promoting resources and live in healthier neighbourhoods, all of which positively impact their health status.

Furthermore, unhindered access to regular health care services is a key determinant of a better quality of life. Among racial minority populations in Canada, inequities/difficulty accessing health care services is a key determinant of reporting lower self-rated health (Garrod et al., 2020). For example, an Ontario-based study revealed that individuals with higher social capital rated their health better compared to those with low social capital scores (Buck-McFadyen et al., 2019). On the whole, social capital can influence an individual’s health in three possible ways: (i) through the promotion of rapid health information in the network, (ii) by increasing the likelihood of the adoption of health behaviours, and (iii) through the use of social control over health-harming behaviours (Kawachi et al., 1999). Finally, geographical factors also influence self-assessed health status through delineating neighbourhood types and characteristics, as well as access to health-enabling resources including proximity to basic health care services and the quality thereof (Caicedo-Velásquez & Restrepo-Méndez, 2020; Saha et al., 2022).

## Study context

Black people constitute about 4.3% (1.5 million) of Canada’s population, but more than half (54.7%) of the country’s Black population resides in the province of Ontario (Statistics Canada, 2021). Toronto, the largest city in Ontario and Canada, is home to more than one third of Black residents in the province and in Canada (Statistics Canada, 2021).

While Ontario, like the rest of Canada, brands itself as a multicultural destination welcoming people of diverse ethno-racial identities, racism remains a stark reality for Black Canadians (Noorishad et al., 2023). In Toronto, for instance, our previous research found that over 66% of Black residents reported personal experiences of racism within the 12 months preceding the study (Husbands et al., 2022). In another study, among Black residents who encountered discrimination, as many as 76% attributed it to their race or ethnicity (The Environics Institute for Survey Research, 2022). Similarly, 35% of Black Torontonians feel that others treat them as inferior, while an additional 25% perceive that they are viewed as unintelligent (The Environics Institute for Survey Research, 2022). These experiences have a noticeable impact on the perceived quality of life among Black people (Paradies et al., 2015). Furthermore, many Black people surveyed perceive structural racism as deeply ingrained in Canada, as their circumstances have failed to garner the concern of policymakers (Matheson et al., 2021). The structural disadvantages suffered by Black Canadians in Ontario are reflected in their heightened levels of poverty, food insecurity, and unemployment/underemployment, all of which are critical social determinants of health (Block et al., 2019; City of Toronto, 2021b).

Additionally, prior studies suggest Black Canadians are overexposed to chronic and infectious diseases (Antabe et al., 2022). In Toronto, for example, Black residents were reported to account for 33% of COVID-19 cases by August 2020 despite making up only 10% of the city’s population (City of Toronto, 2021a). The weSpeak study based in Ontario demonstrated that heterosexual Black men faced many barriers to accessing critical health care services. Black men in the weSpeak study articulated that they felt unwelcome at health care service outlets due to experiences of racism, discrimination, stereotyping, or a complete lack of cultural competence in the services offered (Antabe et al., 2021). While the forgoing observations point to the likelihood of intersectional factors working to impact the quality of life of Black Ontarians, there is a limited research focus on these factors. To address this void, our study aimed to answer the following research question: *What are the key factors associated with Black Ontarians’ quality of self-rated health?*

## Methods

### Design and setting

This paper is based on data from the survey component of the A/C Study^1^ among first- and second-generation Black Canadians aged 15–64 in Toronto and Ottawa. The study protocol has been published elsewhere (Mbuagbaw et al., 2020). Data were collected from November 2018 to December 2019. The study’s protocol was approved by the University of Toronto, Toronto Public Health, the University of Ottawa, and Ottawa Public Health. Participants were informed about the aims and scope of the study and provided informed consent before their participation.

### Sampling and recruitment

Participants were recruited through posters, flyers, and personal contact by peer recruiters at events and venues that cater to Black communities or attract Black patrons. This recruitment strategy has been used widely in prior studies, centred on the Black community in Canada (see Baidoobonso et al., 2016; Konkor et al., 2021) given the challenges and infeasibility of other recruitment methods for this community. Eligibility criteria included individuals who self-identified as African, Caribbean, or Black; were born in sub-Saharan Africa or the Caribbean, or had a parent who was born in these regions; and were capable of communicating in English or French. Sample size was estimated in advance to maintain adequate statistical power based on the planned use of multivariate analyses.

### Data collection

The A/C Study methodology including the data collection procedures has been previously reported (see Mbuagbaw et al., 2020). In brief, participants were invited to complete an electronic survey questionnaire in English or French at the recruitment site or by appointment. The questionnaire was either self-administered or interviewer-assisted on a tablet or laptop and took about 40 min to complete. Each participant received a $40 honorarium. Data were collected on sociodemographic status and individual characteristics including age, education level, employment status, sexual practices, substance misuse/disorder, racism, access to and utilization of health services, and HIV-related care and stigma.

### Measures

#### Outcome

The outcome variable in this study is self-rated health (SRH), which was measured with the global question: “In general, how would you rate your health?” Although subjective, this item has been correlated with objective health indicators and has consistently predicted mortality—often proving more reliable than physician-rated health (Benjamins et al., 2004). Respondents rated their present physical health on a 5-point Likert scale ranging from 1 (excellent) to 5 (poor).

#### Covariate/contextual variables

The covariates (sociodemographic characteristics) that were taken into account in this study include age (< 60 or ≥ 60), sex (male, female, intersex), and education level (< high school, high school, or college or beyond) (see Table 1 for full details). Accessing basic needs and social capital (a relational construct) were evaluated as continuous variables. In our analysis, social capital pertains to the collective understanding, shared norms, established regulations, and interconnected networks that enable a sense of shared community experience within a neighbourhood. The social capital index score is a measurement on a 5-item scale, constructed from individual questions sourced from existing literature. These questions assess the extent of agreement or disagreement regarding individuals’ perceptions of their neighbourhood (Vemuri et al., 2011).

**Table 1:** Sociodemographic characteristics of participants (*n* = 1380)

Characteristic	*n* (%)
Sex*	
Male	492 (36.6)
Female	853 (63.4)
Age*	
< 60 years	1207 (89.7)
≥ 60 years	138 (10.3)
City of residence*	
Toronto	854 (61.9)
Ottawa	526 (38.1)
Education*	
University	739 (55.7)
College	249 (18.8)
High school	309 (23.3)
Less than high school	30 (2.3)
Employment status*	
Not employed	641 (46.4)
Employed part-time	262 (19.0)
Employed full-time	477 (34.6)
Born in Canada (yes)*	298 (22.3)
Experienced racism (yes)	828 (60.0)
Health care access difficulty (yes)*	234 (17.0)
Substance misuse/disorder (yes)^+^	323 (23.4)
Sexual orientation^+^	
Heterosexual	1084 (85.8)
Homosexual	59 (4.7)
Bisexual	87 (6.9)
Other/questioning	34 (2.6)
Meeting basic needs^+^	
Not at all difficult	315 (22.8)
A little difficult	394 (28.8)
Fairly difficult	293 (21.2)
Very difficult	254 (18.4)
Housing situation^+^	
Not adequate	230 (18.3)
Barely adequate	156 (12.4)
Fairly adequate	493 (39.2)
Very adequate	380 (30.2)
Relationship status*	
Single	697 (50.5)
Partner (live apart)	116 (8.4)
Partner (live together)	67 (4.8)
Married	368 (26.7)
Separated/divorced	82 (5.9)
Widowed	19 (1.4)
Self-reported HIV status*	
Positive	75 (8.1)
Negative	846 (91.9)
Self-rated health status	
Excellent	354 (26.6)
Very good	448 (33.6)
Good	371 (27.9)
Fair	110 (8.3)
Poor	22 (1.7)
Characteristic	Mean (SD)^±^
Self-rated health status	3.83 (1.07)
Social capital index score^+^	3.22 (0.72)

*Missing data < 5%, based on total *N*

^+^Missing data 5–10%, based on total *N*

^±^Standard deviation

### Statistical analyses

Data were weighted to reflect the distributions of Black people based on the 2016 Canadian Census data (Statistics Canada, 2019). By combining census and survey data, weights were created by computing the predicted probability of belonging to the survey group adjusting for age, sex, and place of residence. The reciprocal of the estimated probabilities was used to calculate weights. Estimation was done in the presence of missing data.

Sociodemographic variables were expressed in terms of frequencies and percentages for all categorical variables and means and standard deviation for numeric variables. Considering the non-normal distribution of the sample, a generalized linear model (GLM) univariate analysis was used to estimate the associations between SRH as a continuous outcome variable and multiple predictor variables, adjusting for covariates. GLM is an appropriate technique for this analysis, as it generalizes an ordinary regression model. We also evaluated changes in significance and explained variance in independent variables by building a series of linear regression models using an iterative model building approach.

Using GLM univariate analysis, we introduced independent variables one by one in three models. In the first model, we only used demographic factors as independent variables. In the second model, we added health-related factors into the generalized linear regression model based on Model 1. In the third model, we added self-reported social support as an independent variable to Model 2. We determined changes in the significance, parameter size, and variance of SRH caused by each of the independent variables. The variance in SRH caused by each of the independent variables was estimated by *R*^2^. The threshold to denote statistical significance was set at *p* < 0.05. We reported 95% confidence intervals, standard error, and the proportion of missing data for each variable. We examined the independent contributions of predictors of SRH and tested for interaction effects, further described below. The statistical analyses were carried out using the Statistical Package for the Social Sciences, version 28.0 (IBM Corp, Armonk, NY, USA).

## Results

### Sample characteristics

A sample of 1380 self-identified Black people completed the survey (*n* = 828 in Toronto and *n* = 552 in Ottawa) and were included in the analysis. The majority of participants were under the age of 60 (89.7%), female (63.4%), born outside of Canada (75.1%), and residing in Toronto (61.9%). Table 1 illustrates participant demographics and sociodemographic characteristics.

### Predictors of self-rated health

Overall, Black Ontarians reported good health. The mean value for SRH was 3.8 (SD = 1.1) for our sample (1 being excellent and 5 being poor). The majority of respondents perceived their health as very good (33.6%), good (27.9%), or excellent (26.6%). About 8.3% rated their health as fair, and only 1.7% rated their health as poor (see Table 1). In the univariate analysis, the sociodemographic variables of participants, such as sex and sexual orientation, and health-related factors, including difficulties accessing health care, substance misuse/disorder, and meeting basic needs, were the factors that affected their quality of SRH (Table 2).

**Table 2:** Univariate analysis with self-rated health as a dependent variable (*n* = 1380)

Variable	Univariate model	95% CI		*p*-value
	*B* (SE)	Lower	Upper	
Sociodemographics				
Sex	0.226 (0.103)	0.023	0.428	0.029
Age	0.230 (0.190)	− 0.143	0.603	0.226
Education	− 0.471 (0.955)	− 0.2350	1.408	0.622
Employment status	0.051 (0.114)	− 0.173	0.274	0.656
Sexual orientation	0.430 (0.130)	0.174	0.685	0.001
Health-related factors				
Self-reported HIV status	− 0.122 (0.192)	− 0.499	0.255	0.525
Racism	0.084 (0.113)	− 0.138	0.305	0.457
Health care access difficulty	0.327 (0.117)	0.096	0.557	0.006
Substance misuse/disorder	0.210 (0.103)	0.006	0.413	0.043
Meeting basic needs	− 0.125 (0.045)	− 0.213	0.037	0.005
Social support	0.098 (0.068)	− 0.035	0.231	0.148

Self-rated health is used as a continuous variable, with higher scores indicative of poor health

*B* unstandardized coefficient, *SE* standard error, *CI* confidence interval

Lower bound of CI truncated at zero

The influence of demographic factors, health-related factors, and self-reported social support on SRH was analyzed by a three-stage generalized linear model (Table 3). Demographic variables were added in the first model. Being female, older age, and not heterosexual were associated with poorer SRH, while being employed (part-time) was associated with better SRH. In Model 1, our analysis indicated that demographic factors contributed a 6% criterion variance in SRH (*F* = 3.086, *p* < 0.001, *R*^2^ = 0.057). In Model 2, we added a health-related dimension into the analysis based on Model 1 and observed that it accounted for a 14% variance in SRH (*F* = 4.031, *p* < 0.001, *R*^2^ = 0.148). In the second model, health-related factors (health care access difficulty, substance misuse/disorder, and meeting basic needs) were added, where both difficulties accessing health care and substance misuse/disorder were negatively associated with SRH, whereas meeting basic needs was significantly associated with better SRH. In Model 3, we added self-reported social support, which explained 14% of the variance in SRH (*F* = 3.906, *p* < 0.001, *R*^2^ = 0.145), but showed no significant association with SRH. After our final model was constructed, we tested for the effect of the interactions separately and found no significant interaction effects for any of the study variables.

**Table 3:** Generalized linear model analysis with self-rated health as a dependent variable (*n* = 1380)

	Multivariable model 1	Multivariable model 2	Multivariable model 3
	*B* (95% CI)	*B* (95% CI)	*B* (95% CI)
Sociodemographics			
Sex	0.206 (0.088, 0.323)**	0.245 (0.046, 5.854)*	0.226 (0.028, 0.424)*
Age	0.304 (0.118, 0.491)**	0.241 (− 0.127, 0.609)	0.230 (− 0.135, 1.526)
Education	− 0.124 (− 0.521, 0.272)	− 0.301 (− 0.127, 0.609)	− 0.471 (− 0.135, 0.595)
Employment status	− 0.183 (− 0.341, − 0.341)*	0.032 (− 0.188, 0.251)	0.051 (− 0.168, 0.269)
Sexual orientation	0.554 (0.392, 0.716)***	0.453 (0.204, 0.703)***	0.430 (0.180, 0.680)**
Health-related factors			
Self-reported HIV status		− 0.107 (− 0.478, 0.264)	− 0.122 (− 0.491, 0.247)
Racism		0.117 (− 0.095, 0.328)	0.084 (− 0.133, 0.300)
Health care access difficulty		0.354 (0.128, 0.579)**	0.327 (0.101, 0.552)**
Substance misuse/disorder		0.203 (0.004, 0.402)*	0.210 (0.011, 0.408)*
Meeting basic needs		− 0.121 (− 0.206, − 0.036)*	− 0.125 (− 0.211, − 0.039)**
Social support			0.098 (− 0.032, 0.228)
*(Scale)*	1.014^a^ (0.937, 1.097)	0.830^a^ (0.719, 0.958)	0.816^a^ (0.706, 0.942)

*p < 0.05, **p < 0.01, ***p < 0.001

*B* unstandardized coefficient, *SE* standard error, *CI* confidence interval

^a^Maximum likelihood estimate

Self-rated health is used as a continuous variable, with higher scores indicative of poor health

## Discussion

### Overall report of self-rated health

Anti-Black racism in Canada, experienced both structurally and in the everyday interpersonal encounters of Black Canadians, works to undermine their health and well-being. The effects of a persistent structural disadvantage emerge across several indices of health and well-being for the Black community, including income, health, employment, and others. In this paper, we examined the relationships between demographic, socioeconomic, geographic, and health-related factors on the self-reported health status among Black Canadians in Toronto and Ottawa. Notably, this is one of the first known studies to examine these variables, adopting a provincial perspective to analyze the health status and disposition of Black Canadians, in contrast to prior studies that typically have a more localized or regional focus. Our findings show that macro-level factors such as socioeconomic status and comorbidities significantly influenced the quality of life of Black Canadians. While broader studies across ethnically and culturally diverse populations consistently demonstrate that access to health resources and exposure to health risks are influenced by various factors such as age, gender, race, and environmental factors (Gallagher et al., 2016; Schellekens & Ziv, 2020; Singh-Manoux, 2014), our study specifically identifies sex, sexual orientation, access to health care, substance misuse, and meeting basic needs as significant predictors of self-rated health among Black Canadians in Toronto and Ottawa. These findings offer a critical perspective to the emerging scholarship in Canada advocating for a holistic approach to understanding and addressing the health needs of Black people.

We observed that a range of demographic factors predicted the quality of life among our study participants. For instance, biological sex was found to be a key predictor of self-rated health, where women were more likely than men to rate their health as poor. Our finding is consistent with earlier studies, such as Boerma et al. (2016) who attributed this persistent gender gap to biological factors and social dynamics, including gender inequalities that work to expose women to poorer self-assessed health relative to their male counterparts. We also found that people under 60 reported better overall health status compared to those older than 60. Additionally, those with a heterosexual orientation reported a better quality of health/life. This finding is not surprising in the context of entrenched heteronormativity in social interactions and the organization of social services that neglect the needs of sexual minority groups. Coupled with experiences of stigma and homophobia, sexual minorities may be positioned to experience poorer health outcomes (Fredericks et al., 2017; Mule, 2015). A study by Statistics Canada (2021) established a similar pattern where sexual minority status was associated with poorer self-assessed mental health and suicide ideations (Statistics Canada, 2021).

We also found socioeconomic and health-related factors to be associated with self-assessed quality of health. Access to health care was a strong predictor of the quality of self-rated health, where those who have unhindered access to health care gauged their health to be better. This finding underscores the importance of improved access to health care as a critical tool for improving the overall quality of life of Black Canadians. Our findings are consistent with earlier studies in Canada, where Garrod et al. (2020) established that among racialized populations, difficulty in accessing health care was associated with a 12% lower probability of reporting good self-rated health. Not surprisingly, access to basic needs was also found to be a critical determinant of self-assessed quality of life. With this, Black Canadians’ ease of access to the basic necessities of life, including food, clothing, and shelter, was an important factor that influenced their perceived quality of life. Emphasizing the importance of meeting basic needs for health outcomes, Sok et al. (2018) discussed the impact of unmet basic needs on the poor perceived health of people ageing with HIV. Furthermore, substance misuse/disorder emerged as a determinant of the quality of self-rated health/life, indicating that among the Black community in Ontario, the illicit use of substances or misuse tends to influence adversely their experiences and perceptions about the quality of their health. Similar findings have been established in the United States and Brazil, where people who used illicit substances and other coping drugs had a higher likelihood of reporting poor self-rated health (Machado et al., 2017; Mauro et al., 2016).

### Strengths and limitations

The main strength of our study is that it is among the few studies to explore how demographic, socioeconomic, geographic, and health-related factors inform the quality of life of Black Canadians by measuring their self-rated health status. It extends the emerging scholarship on the poor health experiences of Black Canadians and ways in which health policy in Canada can take a holistic approach to addressing this critical challenge. We also acknowledge some study limitations. First, given that our data were collected contemporaneously, our findings are limited to statistical association and should, therefore, be interpreted with caution. Second, evidence indicates that individuals with lower levels of acculturation may downplay the gravity of their health issues or concerns when providing an overall health rating during periods of poor health. As a result, using SRH might conceal existing disparities in such situations and can carry implications for evaluating health disparities within this population. Third, we recognize the limitation of the convenient sample and the venue-based approach to data collection, which may have some implications for the generalizability of findings at the population level. However, this approach has been the most feasible in most research focused on the Black community, given the current challenge of recruiting a representative sample using other approaches (Baidoobonso et al., 2016). Finally, this study was limited to first- and second-generation Black Canadians in Toronto and Ottawa. However, the findings may extend beyond these cities, given that Canada’s Black population is predominantly urban, with the majority being either foreign-born (56%) or having foreign-born parents (35%), mostly from Africa or the Caribbean (90%).

Future studies should explore how demographic, socioeconomic, and health-related factors can be integrated into Canada’s social and health policies to improve the health experiences and outcomes of Black people. Additional studies are needed involving participants from other Canadian provinces, including Black Francophones, and utilizing a longitudinal mixed-methods approach. These studies can explore the meanings behind the subjective assessment of self-rated health and explore the pathways through which the observed demographic and socioeconomic factors impact the health of Black Canadians. Given the COVID-19 pandemic’s disproportionate impact on Black communities, post-pandemic self-rated health data are crucial to capture long-term effects, inform policies, and guide recovery efforts to address health inequities.

## Conclusion

Black Canadians suffer a structural disadvantage that predisposes them to poor health outcomes relative to other Canadians. Yet, we do not know how these structural disadvantages—rooted in demographic, socioeconomic, and health-related factors—impact the quality of their self-rated health. Overall, our findings underscore the importance of improving the social determinants of health as a conduit to enhancing the general health status and the quality of life of Black Canadians. Hence, it is crucial for policymakers in Canada to gain a deeper understanding of the health care and social needs/requirements of Black Canadians. This entails addressing both present challenges and historical barriers that impede their quality of life, through the implementation of tailored social and health policies. Furthermore, positioning this community to meet their basic needs by removing race-based discrimination and structural barriers will go a long way toward helping improve their self-assessed health and quality of life.

## Contributions to knowledge

What does this study add to existing knowledge?

- Findings from this study extend our understanding of the demographic and social factors influencing the self-rated health status and quality of life of Black Canadians.
- A higher proportion of Black Canadians report a “fair” or “poor” self-rated health compared to the national average.
- This is among the first provincial studies to examine the health status and disposition of Black Canadians.
- The findings contribute to the emerging scholarship advocating for a holistic approach to addressing the health needs of Black Canadians.

What are the key implications for public health interventions, practice, or policy?

- There is a persistent need for equitable and tailored policies that address structural and systemic barriers hindering the quality of life and health of Black Canadians, while also improving their social determinants of health.
- An equity-informed approach is crucial for dismantling policies and practices that hinder Black Canadians’ access to quality health care.
- Efforts should concentrate on developing specialized interventions to improve the determinants of health for Black women and Black sexual minorities. Health providers play a pivotal role in this endeavour and must be well trained and equipped to support these efforts.
- Improving the living conditions of Black Canadians is essential to meeting their basic necessities of life.

## Data Availability

Data are available upon request from the Women’s Health in Women’s Hands Community Health Centre.

## Footnotes

1 A/C Study refers to the African/Caribbean track of a series of studies by the Public Health Agency of Canada in collaboration with provincial health departments and community-based organizations. The purpose of the A/C study is to develop an understanding of underlying factors that augment the HIV risk and vulnerability of Black communities and to inform policy and practice in Ontario, Canada with regard to HIV care for Black people.
